# Five weeks of intermittent transcutaneous vagus nerve stimulation shape neural networks: a machine learning approach

**DOI:** 10.1007/s11682-021-00572-y

**Published:** 2021-12-29

**Authors:** Martina. A. Obst, Arkan Al-Zubaidi, Marcus Heldmann, Janis Marc Nolde, Nick Blümel, Swantje Kannenberg, Thomas F. Münte

**Affiliations:** 1 Department of Neurology, University of Lübeck, Lübeck, Germany; 2 Applied Neurocognitive Psychology Lab, University of Oldenburg, Oldenburg, Germany; 3 University of Western Australia, Perth, Australia; 4 Department of Internal Medicine 1, University of Lübeck, Lübeck, Germany; 5 Centre of Brain, Behavior and Metabolism (CBBM), Universität of Lübeck, Building 66 Ratzeburger Allee 160, 23562 Lübeck, Germany

**Keywords:** tVNS, Obesity, Human, rs- fMRI, Reward, Saliency, Interoception, Machine learning classification, fALFF

## Abstract

Invasive and transcutaneous vagus nerve stimulation [(t)-VNS] have been used to treat epilepsy, depression and migraine and has also shown effects on metabolism and body weight. To what extent this treatment shapes neural networks and how such network changes might be related to treatment effects is currently unclear. Using a pre-post mixed study design, we applied either a tVNS or sham stimulation (5 h/week) in 34 overweight male participants in the context of a study designed to assess effects of tVNS on body weight and metabolic and cognitive parameters resting state (rs) fMRI was measured about 12 h after the last stimulation period. Support vector machine (SVM) classification was applied to fractional amplitude low-frequency fluctuations (fALFF) on established rs-networks. All classification results were controlled for random effects and overfitting. Finally, we calculated multiple regressions between the classification results and reported food craving. We found a classification accuracy (CA) of 79 % in a subset of four brainstem regions suggesting that tVNS leads to lasting changes in brain networks. Five of eight salience network regions yielded 76,5 % CA. Our study shows tVNS’ post-stimulation effects on fALFF in the salience rs-network. More detailed investigations of this effect and their relationship with food intake seem reasonable for future studies.

## Introduction

The vagus nerve is a key structure connecting the brain with the internal organs. It plays an important role in the control of food intake (de Lartigue, 2016). Its central relay nucleus, the nucleus tractus solitarius (NTS, Kalia & Sullivan, 1982) is connected with multiple subcortical and – via polysynaptic pathways – cortical regions (Henry, 2002). Because of this anatomical arrangement, the vagus nerve has been considered as a target for neuromodulatory therapy of conditions like epilepsy, depression and migraine starting in the ninth decade of the last century (Penry & Dean, 1990; Rutecki, 1990; Sadler et al., 2002). Indeed, a number of controlled studies have demonstrated the efficacy of vagus nerve stimulation (VNS) in epilepsy (e.g. Wheless et al., 2018) and depression (e.g. Lv et al., 2019). Interestingly, invasive VNS has been shown to lead to loss of weight (Banni et al., 2012; Bugajski et al., 2007; Burneo et al., 2002; Gil et al., 2011; Pardo et al., 2007; Vijgen et al., 2013), in particular in patients with elevated BMI, raising the possibility to use VNS as an adjunct treatment of obesity (Göbel et al., 2017, Val-Laillet et al., 2015).

A newer development, making VNS potentially more accessible as a treatment, has been transcutaneous VNS (tVNS), that takes advantage of the fact that the auricular branch of the vagus nerve can be stimulated at the outer ear canal. TVNS has been shown to modulate widespread networks of brain regions (Badran et al., 2019; Frangos et al., 2015; Yakunina et al., 2017, 2018) including NTS, bilateral spinal trigeminal nucleus, dorsal raphe, locus coeruleus, contralateral parabrachial area, amygdala, hippocampus, hypothalamus and nucleus accumbens as well as cortical sites. Recent evidence from our group also suggests that tVNS applied in a single 30 min session prior to an fMRI scan is able to change responses to food-related stimuli (Alicart et al., 2020) .

What is not known, however, is whether tVNS applied intermittently for several hours per day over several weeks is shaping spontaneous brain activity. Such an alteration of spontaneous activity in brain networks might provide the basis for the efficacy of tVNS in conditions like epilepsy, depression, migraine and, possibly, obesity. To expect such adjustments of resting state (rs) network activity and connectivity is not unreasonable, as repetitive training or only one session of a novel task can induce persisting reorganization of resting state networks (RSNs). This has been shown for different cognitive domains, including memory (Dresler et al., 2017), language (Waites et al., 2005) and visual perception (Guidotti et al., 2015; Urner et al., 2013).

The present report focusses on tVNS effects on rs-neural activity. It is part of a larger dataset that investigated tVNS’ potential as a treatment option for obesity. To this end we carried out an experiment during which obese participants either received tVNS or sham stimulation for several hours per day over a period of 5 weeks. Further results regarding effects on blood hormones of metabolism, the processing of visual food-stimuli and reward associated behavior will be reported elsewhere.

One way to demonstrate effects outlasting the actual stimulation is by application of machine learning techniques to rs activity, i.e. fractional amplitudes of low frequency fluctuations (fALFF). FALFF represents the local spontaneous fluctuation of the fMRI BOLD signal (Zou et al., 2008) suitable to gain information about the magnitude of a brain region’s activity. The advantage of fALFF is its high temporal stability (Küblböck et al., 2014) and test-retest reliability (Zou et al., 2008). A multivariate pattern analysis (MVPA) was conducted on the group level to search for a pattern in the data (classifier, subset of given features) that differentiates between the stimulation groups best. For the analysis we utilized a sequential floating forward selection (SFFS) strategy in conjunction with a linear support vector machine (SVM) classification and leave one out cross-validation (LOOCV). In particular the combination of feature-selection algorithms (to remove redundant information) with a machine learning classification method (that are less sensitive to a high dimensionality such as linear SVM) and a cross-validation procedure (that evaluates the classification accuracy and generalizability for unseen data) has been recommended to avoid the issue of overfitting and to improve generalizability of the results (Al-Zubaidi et al., 2019; Farooq & Hussain, 2016; Kukolja et al., 2014; Tang et al., 2006). Next, the results were controlled for random effects by applying random permutation tests (Al-Zubaidi et al., 2019; Pereira et al., 2009). Beside the traditional permutation procedure, we conducted a second control that includes the feature selection process. Our second control allows an estimate of overfitting and ensures better generalizability of the CA results.

A reliable classifier would suggest the presence of long-lasting tVNS effects on rs brain activity. Finally, to explore the potential significance of the network changes in the context of the larger study, we analyzed the relation of these patterns (subsets) with the perceived hungriness and satiety within each participant.

## Methods

### Subjects

All procedures had been approved by the ethical committee of the University of Lübeck. Thirty-four male participants gave written informed consent to participate. All participants were obese (BMI, WHR), had no clinical depression disorder (BDI) and an unremarkable eating behaviour (FEV questionnaire) (Table 1). The groups (tVNS vs. sham stimulation) did not differ regarding these variables at the baseline level (pre-intervention). Proofed via blood samples only those applicants were included into the trial without any metabolic disorder (i.e., diabetes, metabolic syndrome). Further exclusion criteria, assessed by a short medical history, comprised particular eating habits (vegetarian or vegan diet), food intolerances, the current inclusion in any weight reduction programmes, smoking, consumption of drugs, an arrhythmic sleeping behaviour (sleep disorder, shift working), regular medication intake, any major somatic or psychological disorder in the present or in the past and implanted or unremovable metallic objects (peacemaker, prosthesis). All subjects had normal or corrected-to-normal vision and were naive regarding vagus nerve stimulation.

**Table.1:** Descriptive sample statistics

n = 17 per group	tVNS Mean (SD)		sham Mean (SD)		statistic	
Age	30.53	(5.83)	34.00	(7.75)	t(32) = -1.48	p = 0.150
BMI	34.52	(6.49)	34.02	(5.43)	z = 0.34	p = 0.731
WHR	1.0213	(0.06)	1.001	(0.06)	z = 1.14	p = 0.256
BDI	3.2941	(2.76)	1.94	(2.38)	z = 1.72	p = 0.086
FEV CC	6.88	(4.00)	7.88	(3.86)	z = 0.78	p = 0.436
FEV DE	6.12	(3.64)	7.94	(3.99)	z = 1.35	p = 0.177
FEV HF	4.47	(3.83)	5.77	(3.40)	z = 1.25	p = 0.212
Stim. duration (h/day)	3.80	(0.2)	3.8	(0.2)	w = 126	p = 0.766
Stim. intensity (mA)	1.4	(0.6)	1.3	(0.5)	w = 139	p = 0.922

Abbreviations: BMI: Body Mass Index, kg/m2; WHR: Waist-Hipp-Ratio; BDI: Beck Depression Inventory, FEV: German Questionnaire for Eating behavior, subscale CC = cognitive control, DE = disturbability of eating, HF = hunger feelings.

t = two-sided two sample t-test; z = two-sided Mann-Whitney U-test

### Procedure

**Fig. 1:**
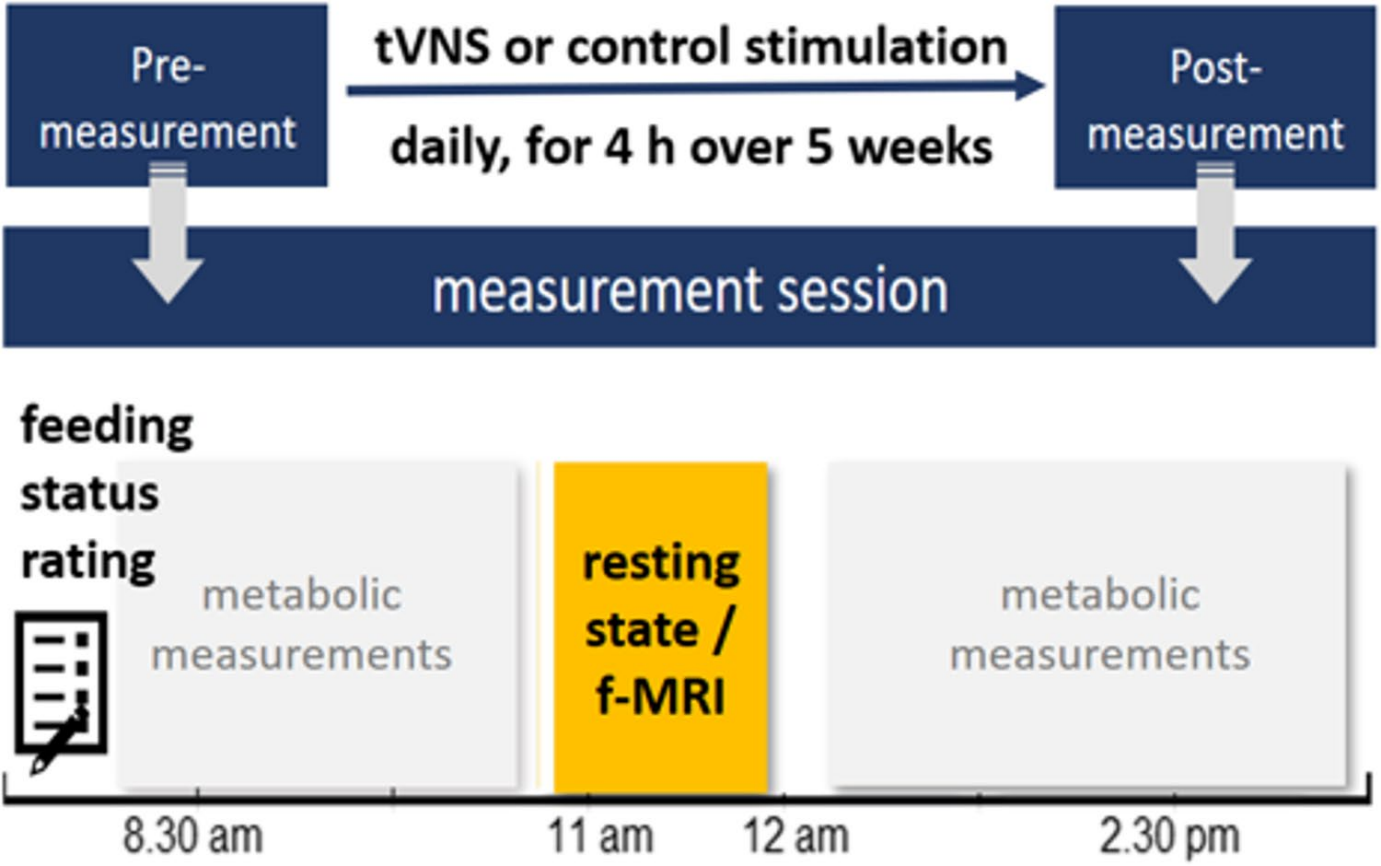
Experimental design

Participants were assigned to either the experimental (tVNS) or control group (sham stimulation). In a pre-post study design, the participants took part in two measurement sessions - once before the treatment was given and once after it. Before both sessions, participants were obliged to fast from 6 pm the day before to achieve a comparable baseline in terms of food metabolism (i.e., blood glucose level) and the associated hunger or satiety status. As visible in Fig. 1, both sessions started at 8.30 h with answering questionnaires (among those the VAS for rating the hunger and satiety status), followed by several metabolic investigations (results will be published in a forthcoming article). At approx. 11 a.m. the rs-fMRI measurement was conducted. At the end of the first session, the stimulation device and its application (in respect to the treatment condition) were instructed to the participants and the device was handed out. All participants self-administered the stimulation at home over five weeks for 4 h each day. Participants were asked to keep a diary, noting the daily stimulation time, -duration and -intensity. Additionally, they were supervised by the experimenters through weekly phone calls to ensure an unproblematic stimulation application.

### tVNS and control stimulation

**Fig. 2:**
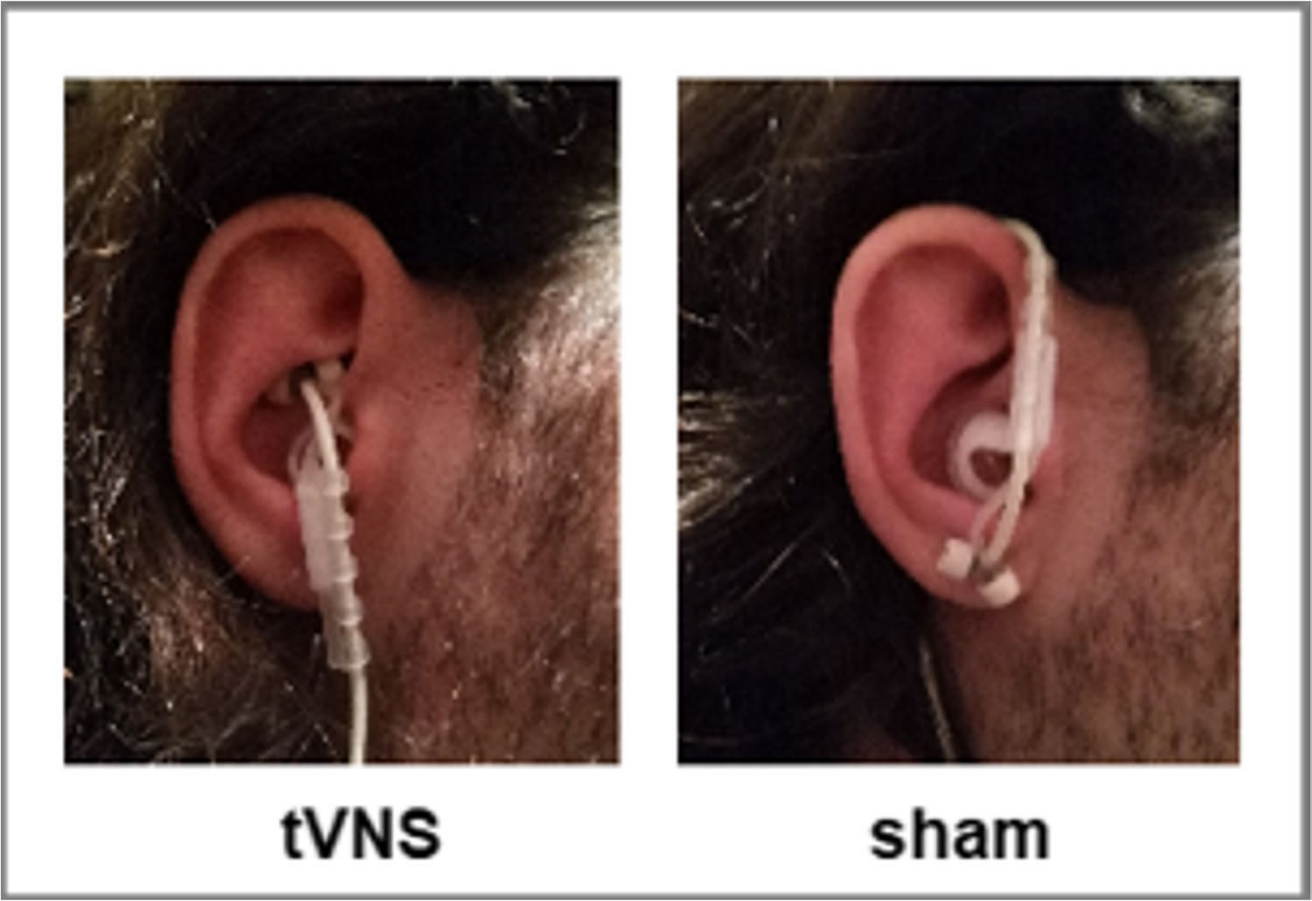
Electrode positions (NEMOs® Cerbomed Erlangen-Germany). Right) Transcutaneous stimulation of the auricular branch of the vagus nerve (tVNS, experimental condition) of the left ear, Left) stimulation of the left earlobe (control condition). Stimulation parameters: 25 Hz, biphasic, 30 s ON and 30 s OFF interval, current intensity was adjusted individually until a tingling was felt

The NEMOS device (tVNS Technologies GmbH ©) was used which consists of a stimulation unit and an ear electrode (Fig. 2). In the tVNS condition the electrode was placed at the cymba conchae of the left ear that stimulated the auricular branch of the vagus nerve. In the control condition, the vagus nerve was not stimulated by placing the electrode at the left earlobe (Berthoud & Neuhuber, 2000). In both conditions the stimulation was performed with a frequency of 25 Hz, a biphasic impulse interval (30 s on, 30 s off). The stimulation intensity was adjusted individually so that participants perceived a tingling but no pain constantly across the entire daily application period. The device was preprogrammed for 4 h of daily stimulation. It showed the test subjects via a bar how much of it they had already achieved. In addition, the device saved in a stimulation history how much the stimulation was administered the day before, in the last month and in the last 3 months (i.e., yesterday: 100 %, 1 month: 97 %, 3 months: 95 %). In addition, the device ensured an accurate treatment application by stopping the stimulation in case of i.e., a contact loss of the electrode or if it was broken (this causes a repeated beep tone to be heard). In this case, no data is saved in the stimulation history. We also verified the treatment success by specifically examining pre-post effects in brainstem regions which were reported with the tVNS treatment previously.

The total duration of stimulation was similar in both groups (VNS M 95 % +- 5.5 % ; Sham 96 % +- 6 %) as well as the stimulation intensity (VNS M 1.1 mA +-0.6mA ; Sham M 1.2 mA +- 0.5 mA) (Table 1).

### rs-fMRI image acquisition

Structural and functional MR imaging was performed using a 3-T Siemens Magnetom Skyra scanner with a 64-channel head-coil. Blood oxygen level dependent (BOLD) signal within the resting state (eyes open) was acquired by a T2*-weighted single-shot gradient-recalled echo-planar imaging (GRE-EPI) sequence (TR = 1.5 ms, TE = 25, flip angle = 70°, in-plane resolution 3 × 3 × 3 mm, whole brain volumes = 320, FOV = 512 × 512, GRAPPA factor 2,simultaneous multi-slice factor 2). Participants were instructed to lie motionless inside the scanner and to fixate a white cross on a black screen (visual angle 20°). They were instructed to let their thoughts wander during and to not think of anything specific during the measurement-period of eight minutes. Additionally, structural images of the whole brain were acquired by using a 3D T1-weighted MP-RAGE scanning sequence (TR = 1 ms, TE = 2.44, TI = 900, Flip angle = 9°, Res = 1 × 1 × 1 mm, FOV = 256 × 256; acquisition time = 4.5 min).

### Data pre-processing

Rs-fMRI data were pre-processed with the DPARSFS toolbox (Version 4.5, Yan & Zang, 2010), a toolbox within DPABI (Version 4.1; Yan et al., 2016) that is based on Statistical Parameter mapping (SPM12). Both run under Matlab. The pre-processing comprised the following steps:

- (i) removal of the first 10 volumes (ii) slice-timing correction (iii) images realignment (iv) co-registration of the mean functional image with T1 image (v) images segmentation into grey matter (GM) ,white matter (WM), and cerebrospinal fluid (CSF) (vi) bias correction of functional images (vii) spatial normalization and adjustment to the standard MNI-space of T1-images with DARTL algorithm (Ashburner, 2007), (viii) noise reduction (of undefined physiological effects) with nuisance-regression for WM and CSF (Teeple et al., 2018), (ix) spatially normalization to MNI space of the functional images by using DARTL with a 3 × 3 × 3 voxel size (x) smoothing with a 6 mm full-width at half maximum (FWHM) Gaussian-kernel.

### Classification analysis

In a first step, we performed a classification analysis with specific literature-based brainstem regions (see section Regions of interest (ROI Extraction)) to test whether the application of the stimulation was successful (positive control). Next, a classification analysis on the entire brain was conducted to assess the most informative brain regions for differentiating between tVNS and control group. In a third step we tested hypothesis-driven for treatment effects on RSNs. In the context of the current experiment it was asked whether tVNS effects on food intake were driven by stimulation-dependent alterations within the reward RSN (Wijngaarden et al., 2015; Zhang et al., 2015). Following up recent work of our group (Obst et al., 2020) we also tested for effects on the primary visual (VIS), salience (SAL), executive control (CE) and dorsal attentional (DA) networks.

### Fractional amplitude of low-frequency fluctuation (fALFF)

We used the DPARSFS toolbox for the calculation of fALFF values. Subject specific fALFF maps were calculated by performing a fast Fourier transform (FFT) converting the data from the time domain (not temporal band-pass filtered) to the frequency domain to obtain the power spectrum for each voxel. The power spectrum was square-rooted to estimate the amplitudes of each frequency and afterwards filtered of the low-frequencies (0.01–0.08 Hz), resulting in ALFF. By averaging the low frequencies and dividing through the entire frequency range we obtained fALFF. Subsequently, individual fALFF maps were transformed to z-scores (zfALFF) to improve the comparability of the different brain regions of interest. For the brainstem and exploratory whole brain analysis, zfALFF was calculated for the entire brain (default mask). Here, subsequent analysis were restricted to grey matter by the applied ROIs derived from the AAL atlas (see section *ROI extraction*). In contrast, in the network analysis zfALFF was only calculated directly within a grey matter mask to avoid white matter signal extraction from the manually built ROIs. The grey matter mask was constructed by multiplying a sample grey matter mean-mask with the grey matter mask of the AAL atlas. Finally, the difference between pre- and post-treatment zfALFF maps (Post-Pre) was calculated for each participant. Positive difference values therefore embody an increase in brain activity over the time and the results of subsequent analysis reflect an interaction with this time factor.

### Regions of interest (ROI) extraction

For all analyses, zfALFF values were extracted with Marsbar (version 4.4, Brett et al., 2002) from the analysis-relevant brain regions. The whole brain analysis comprised 127 ROIs including all regions of the automated anatomical labelling atlas (AAL with cerebellum) and additional brainstem regions which were taken from the Harvard Ascending Arousal Network Atlas (AAN; including dorsal raphe [DR], locus coeruleus [LC], median raphe [MR], parabrachial complex [PBA], peduncular pontine nucleus [PP] and ventral tegmental area [VTA]). For the brainstem analysis 13 ROIs were chosen based on the results of Frangos et al. (2015). The ROI-space for classification comprised the red nucleus (RN) and substantia nigra (SN), both taken from the AAL atlas, as well as the DR, LC, the periaqueductal grey (PAG), the PBA and VTA, which were all taken from the AAN. We additionally built ROI-masks with a radius of 3 mm for the left and right spinal trigeminal nuclei (sTN, MNI coordinates: ± 2, -44, -62, Lerebours et al., 2019) and the nucleus tractus solitary (NTS, MNI coordinates: ± 8, -38, -42, Garcia et al., 2017) in Marsbar. The RSN ROIs were built in Marsbar along the specifications of Razi et al. (2017), Wijngaarden et al. (2015) and Zhang et al. (2015; see Table 2 for MNI coordinates) with a radius of 6 mm.

**Table.2:** MNI coordinates of tested resting state (rs) networks

Rs-Networks	ROIs	X	Y	Z
Salience (SAL)	dACC	± 5	14	42
	aPFC	+ 32 / -35	45	30
	aINS	± 32	16	6
	LPC	± 62	-45	30
Reward (REW)	dACC	± 5	14	42
	aINS	± 32	16	6
	L-AMYG	21.4	-0.3	-18.8
	r-AMYG	-25.1	-0.2	-18.5
	HYPO	5 /-4	-1	-13
	L-NAcc	-8.8	13.2	-7.8
	r-NAcc	11.6	13.3	-7.7
	Caudate ncl.	-10 / 11	13	9.5
	Putamen	± 23	-2	3
	L-vStr	-14.6	11.4	-5.4
	r-vStr	21.9	15.3	-1.3
Dorsal Attention (DA)	FEF	± 29	-9	54
	IPS	± 26	-66	48
	aIPS	41 / -44	-39	45
	L-MTG	-50	-66	-6
	r-MTG	53	-63	-6
Control Executive (CE)	dmPFC	0	24	46
	aPFC	± 44	45	0
	SPG	± 50	-51	45
Visual (VIS)	l-V1	-11	− 81	7
	r-V1	11	-78	9
	V2	29 / -19	-92	2
	L-V3	-45	-75	11
	r-V3	44	-75	5

Abbreviations according to AAL atlas; aPFC = anterior prefrontal cortex, LPC = lateral parietal cortex, HYPO = Hypothalamus, vSTr = ventral striatum, FEF = frontal eye field,IPS = inferior parietal sulcus, dmPFC = dorsomedial PFC, V1-V3 = visual cortex 1–3.

### Machine Learning - Support vector machine (SVM) classification

For all analyses (see section Classification analysis) classification was implemented with a linear support vector machine (SVM). SVM classification is a mathematical pattern recognition procedure that tries to find a line (for two dimensional) or a hyperplane (for manifold dimensional data) that splits the data into two classes (e.g., tVNS vs. sham group, Vapnik, 1998). The hyperplane is defined by chosen data features (that are in our case the selected brain regions). Similar to logistic regression, the SVM tries to predict a categorical dependent variable (DV, here tVNS or control) by metrical independent variables (IV, selected brain regions). However, while in logistic regression this function is found by minimizing the prediction error of all data points, SVM builds this function by finding the largest margin between those data points on the border between the classes. SVM classification has been shown to be robust with small sample sizes (Vabalas et al., 2019). The search for this function consists of two steps: First the training step, where the class-membership is known, and the classifier is built in dependence of the amount of correct class allocations. In a second validation step (see section *leave one out cross validation*), the hyperplane is tested by predicting the class of unseen data.

### Leave one out cross validation (LOOCV)

The test-training procedure was conducted with LOOCV. In several loops the classifier is trained on n-1 datapoints. Subsequently, the classifier is tested on the excluded subject. After running this procedure for each subject, the classification accuracy (CA) is calculated based on correct and wrong predictions (∑ right predictions / number of tests). For the goal of estimating the prediction error (or in other words estimating the CA) Zhang and Yang (2015) demonstrated the suitability of LOOCV (compared to other k-fold CV methods) even if the single parameters are correlated. Moreover, to clarify the quality of the hyperplane (subset) we additionally calculated the sensitivity (true positive), the specificity (true negative) and the diagnostic odds ratio (DOR) to gain information about the quality of the validation procedure.

### Sequential forward floating selection (SFFS)

To optimize the search for the best hyperplane (subset), we applied the sequential forward floating selection algorithm (SFFS; Pudil et al., 1994, Burrell et al., 2007). The goal of the SFFS algorithm is to find the subset out of all given features – which are several ROIs in our case - that classifies best between the groups. The algorithm starts with finding the feature with the highest classification weight (lent during the SVM classification procedure) and keeps it in a candidate subset. Subsequently, the second-best feature is searched and added to the first in a new candidate subset and so on. All identified subsets are thereupon subjected to the training-testing procedure (LOOCV) and the final CA is calculated enabling to determine the best subset that is the final hyperplane. The benefit of the SFFS algorithm is its ability to re-evaluate previously selected features and to remove them from the candidate subset if this improves the classification accuracy. Therefore, SFFS is less prone to the nesting problem and more sensitive to find the best subset (Pudil et al., 1994). Feature selection is most relevant in our second, whole brain analysis (see section analysis) asking for the most important ROIs in the entire brain to separate between tVNS and sham condition. Here, the most informative result is embodied by the smallest subset with the highest CA.

### Control permutation

To test whether the experimental subset is more than just a random artifact and whether our initial data set contains more information than just noise, we conducted two different control permutations. The traditional control tests the performance of the experimental subset on a dataset with randomized group labels for 10,000 times (Al-Zubaidi et al., 2019; Golland and Fischl 2003; Pereira et al., 2009). Here, the mean CA should be around 50 % reflecting the null distribution of accuracy based on resampling. However, this procedure does not control how likely it is to find any subset of the same length in randomized data that classifies it comparably well – an important point for the generalizability of the experimental subset. Therefore, we included the feature selection process in the permutation procedure (SFFS, SVM and LOOCV) in our second control (Feature Selection Control, FSC). Since the SFFS algorithm is designed to find a subset that classifies with a high accuracy, the FSC is much more conservative than the traditional one and mean CAs above 50 % are to be expected.

Due to the very high computational costs involved in this second control, we estimated the number of permutations (proportion estimation) required a priori. In order to be able to reliably detect a difference of at least 10 %, at least 400 permutations must be carried out according to the formula n = 4 / W ^ 2 (where n = number of permutations and W = the difference between the data). To be on the safe side, we carried out the FSC with 500 runs.

Controlling the feature selection step as well has the benefit to get confidence about the reliability of the experimental subset and the classification accuracy. It also enables to estimate the amount of possible overfitting. Data overfitting is an important problem in machine learning analysis and means that the machine learns the classification pattern based on noise instead of real information; overfitting is therefore related to the size of the given feature pool and affects the generalizability of the classification result. The extent of overfitting is represented by the difference between chance level (50 %) and the actual reached CA of the permutation. For example, if the permutation achieves a CA of 80 %, then there is probably an overfitting of 30 %.

In both controls, to prevent the random relabelling from generating a mostly reversed original dataset by chance, only a random half of the participants in each group were assigned with the opposite label.

Finally, for both controls, the probability (p) of the experimental CA being smaller or equal to the average control CA was calculated (Golland & Fischl, 2003). P-values smaller than 0.05 were defined as significant. We also report Cohens d_z_ for the FSC-significances.

### Behavioral relations – Regression analysis

We used multiple regression analysis to investigate the relationship between the activity within significant experimental subset-ROIs (independent variables) on perceived hungriness and satiety (dependent variables). Perceived hungriness and satiety were measured via a visual analogue scale (14 cm). Again, the time difference between pre-and post-ratings (post- pre) was calculated and therefore, positive difference values represent an increase in either hungriness or satiety. Given the assumption that not all brain regions of a subset would be relevant to predict the perceived nutritional status, we conducted stepwise regression modelling. To test for treatment effects the interaction of each ROI with the group factor (tVNS vs. sham) was included. The regression modelling was carried out in R (version 3.6.0). All resulting regression models meet assumption criteria of regression and significance testing was conducted at the 0.05 alpha level.

## Results

### Brainstem analysis – proof of treatment effect

**Fig. 3:**
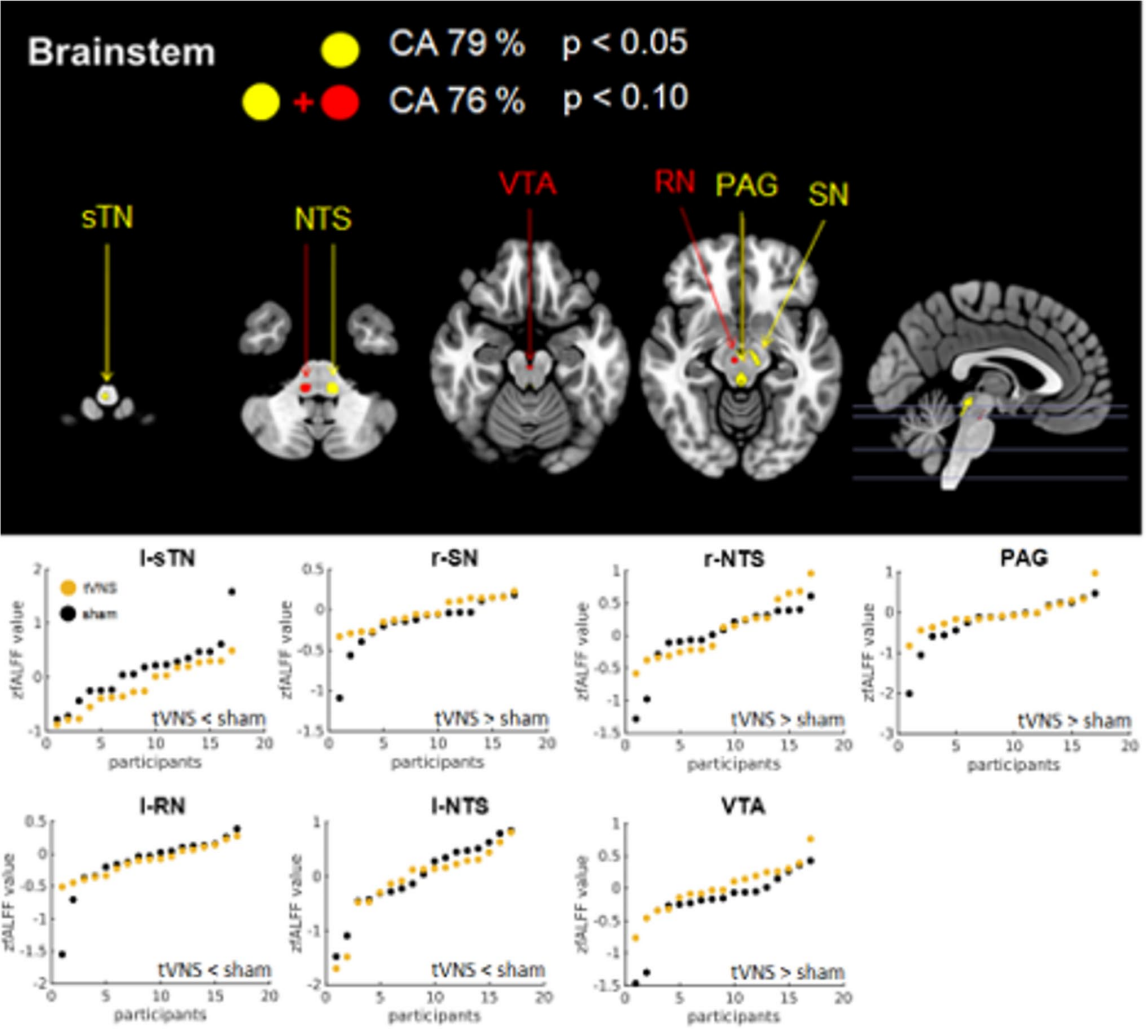
Classification result of the brainstem analysis. Upper panel: Shows the network of regions of interest (ROIs) selected by sequential forward floating selection algorithm (SFFS) out of 13 ROIs reaching a classification accuracy (CA) of 79 % by utilizing support vector machine (SVM) algorithm; p describes the probability that the classification result statistically equals the classification result of the control permutation (significance test). Lower panel: Visualization of mean zfALFF values and group differences per ROI and stimulation group (hierarchical ascending ordered from top-left to bottom-right): l = left, r = right, spinal trigeminal nucleus (sTN), nucleus of solitary tract (NTS), substantia nigra (SN), periaqueductal grey (PAG), red nucleus (RN), ventral tegmental area (VTA)

Using 13 brainstem ROIs for classification a CA of 79.4 % was reached due to the l-sTN, the r-SN, the r-NTS and the PAG (hierarchical order, see Fig. 3). The additional inclusion of the l-RN, the l-NTS and the VTA yielded a CA of 76.5 %. Both subsets showed a good detection rate as indicated by the sensitivity (> 0.8) and specificity (> 0.7) values (Table 3.a). While both permutation tests were significant for the smaller subset (Table 4.a), the larger subset only showed a significant result in the traditional control and a trend effect in the FSC. Nevertheless, Cohens d_z_ points to a very large effect for both subsets. However, tVNS activated all of these regions except the l-sTN, the l-RN and the l-NTS which showed a reduced activity in the tVNS condition (see Table 5.A).

**Table.3:** Characteristic values of classification analysis

	Analysis	feature ratio	experimental CA (%)	Sens| Spec | BA
verification analysis				
a1	Brainstem	4/13	79.41	0.92 | 0.73 | 0.83
		7/13	76.47	0.85 | 0.71 | 0.78
		13/13	55.88	
exploratory analysis				
b1	Whole Brain	9/127	94.12	1.00 | 0.90 | 0.95
hypothesis driven analysis				
c1	SAL	5/8	76.47	0.85 | 0.71 | 0.78
		6/8	73.53	0.83 | 0.68 | 0.76
		7/8	67.65	0.75 | 0.64 | 0.70
		8/8	61.76	
c2	DA	4/8	70.59	0.65 | 0.82 | 0.74
		8/8	23.53	
c3	REW	8/17	67.65	0.75 | 0.64 | 0.70
		17/17	52.94	
c4	VIS	3/6	67.65	0.64 | 0.75 | 0.70
		6/6	50.00	
c5	CE	3/5	61.76	0.61 | 0.63 | 0.62
		5/5	55.88	

Classification of groups (tVNS vs. Sham) was conducted on resting state fMRI zfALFF values by using SFFS algorithm and SVM

CA = Classification Accuracy. Sens |Spec | BA = sensitivity, specificity, and balanced accuracy

**Table.4:** Characteristic values of control permutation analysis

	Analysis	ratio	exp.	permutation control CA (%)				
			CA (%)	traditional (10,000 permutations)		FSC (500 permutations)		
				mean (SD)	p	mean (SD)	p	*d*_*z*_
verification analysis								
a	Brainstem	4/13	79.41	49.54 (0.12)	0.003**	71.68 (5.8)	0.044 *	**1.33**
		7/13	76.47	49.46 (0.13)	0.014*	67.83 (8.1)	0.086 °	1.07
		13/13	55.88					
exploratory analysis								
b	Whole Brain	9/127	94.12	49.82 (0.13)	< 0.001***	85.16 (6.5)	0.044 *	**1.38**
hypothesis driven analysis								
c1	SAL	5/8	76.47	49.69 (0.16)	0.010*	65.35 (8.1)	0.048 *	**1.38**
		6/8	73.53	49.92 (0.13)	0.030*	62.57 (9.3)	0.086 °	1.17
		7/8	67.65	49.76 (0.13)	0.094°	56.81 (11.0)	0.130	0.98
		8/8	61.76	49.58 (0.13)	0.207	48.46 (12.6)	0.132	1.05
c2	DA	4/8	70.59	49.80 (0.13)	0.046*	65.51 (8.2)	0.210	0.62
		8/8	23.53	49.73 (0.13)	0.084°			
c3	REW	8/17	67.65	49.55 (0.13)	0.094°	72.87 (8.1)	0.686	-0.64
		17/17	52.94					
c4	VIS	3/6	67.65	49.41 (0.12)	0.064°	62.63 (8.3)	0.214	0.60
		6/6	50.00					
c5	CE	3/5	61.76	49.69 (1.12)	0.120	60.69 (9.0)	0.390	0.11
		5/5	55.88					

Exp-CA = experimental classification accuracy; traditional permutaion control as described in i.e., Pereira et al., 2009; FSC = feature selection control; our improved permutation control checking for overfitting,

p = probability of the true classification accuracy being part of to the empirical distribution of the random permutation (see Golland & Fischl, 2003); n = 10,000 (traditional) or 500 (FSC) with alpha < 0.05. Effect size Cohens d_z_ calculated for the difference between experimental CA and FSC result

Signif. codes: *** p < 0.001, ** p < 0.01, * p < 0.05, ° p < 0.1 (trend effect)

**Table.5:** Descriptive statistic of the Whole Brain, Brainstem and Salience network analysis

A. Brainstem			B. Whole Brain			C. Salience			
	tVNS	sham		tVNS	sham		tVNS		sham
**l-sTN**	-0.27 (0.7)	0.18 (0.6)	**l-REC**	-0.01 (0.1)	0.13 (0.4)	**l-aPFC**		0.16 (0.3)	-0.10 (0.5)
**r-SN**	-0.06 (0.3)	-0.07 (0.2)	**l-CER6**	0.04 (0.4)	-0.03 (0.3)	**r-aPFC**		0.05 (0.5)	-0.03 (0.4)
**r-NTS**	0.12 (0.6)	0.08 (0.4)	**r-CER6**	0.03 (0.2)	-0.02 (0.2)	**l-aINS**		-0.06 (0.1)	-0.10 (0.3)
**PAG**	-0.10 (0.4)	-0.11 (0.7)	**RapheD**	0.06 (0.3)	-0.19 (0.4)	**r-aINS**		-0.01 (0.3)	-0.10 (0.4)
**l-RN**	-0.09 (0.4)	-0.04 (0.4)	**l-AMYG**	0.04 (0.3)	0.07 (0.5)	**l-dACC**		0.13 (0.3)	0.17 (0.3)
**l-NTS**	-0.13 (0.6)	-0.04 (0.8)	**l-SFGmedial**	0.09 (0.3)	0.12 (0.3)	**r-dACC**	0.10 (0.42)		0.10 (0.36)
**VTA**	-0.03 (0.4)	-0.16 (0.3)	**r-SFGmedial**	0.15 (0.3)	-0.03 (0.4)				
			**l-ANG**	-0.19 (0.5)	-0.06 (0.3)				
			**r-CAL**	-0.05 (0.4)	-0.02 (0.5)				

Reported are Median (IQR) for zfALFF values of ROIs

Abbr. according to AAN atlas(for brainstem structures) and AAL atlas (for cortical and subcortical structures): sTN =, SN = substantia nigra, NTS = nucleus of solitary tract, PAG, periaqueductal grey, RN = red nucleus, VTA = ventral tegmental area, CER6 = cerebellum lobule 6, DR = dorsal raphe nucleus, SFGmedial = superior medial frontal gyrus, CAL = Calcarine Sulcus, aPFC = anterior Prefrontal cortex, dACC = dorsal anterior cingulate cortex, aINS = anterior insula; l = left, r = right.

### Whole brain analysis (127 ROIs) – test for most affected regions

**Fig. 4:**
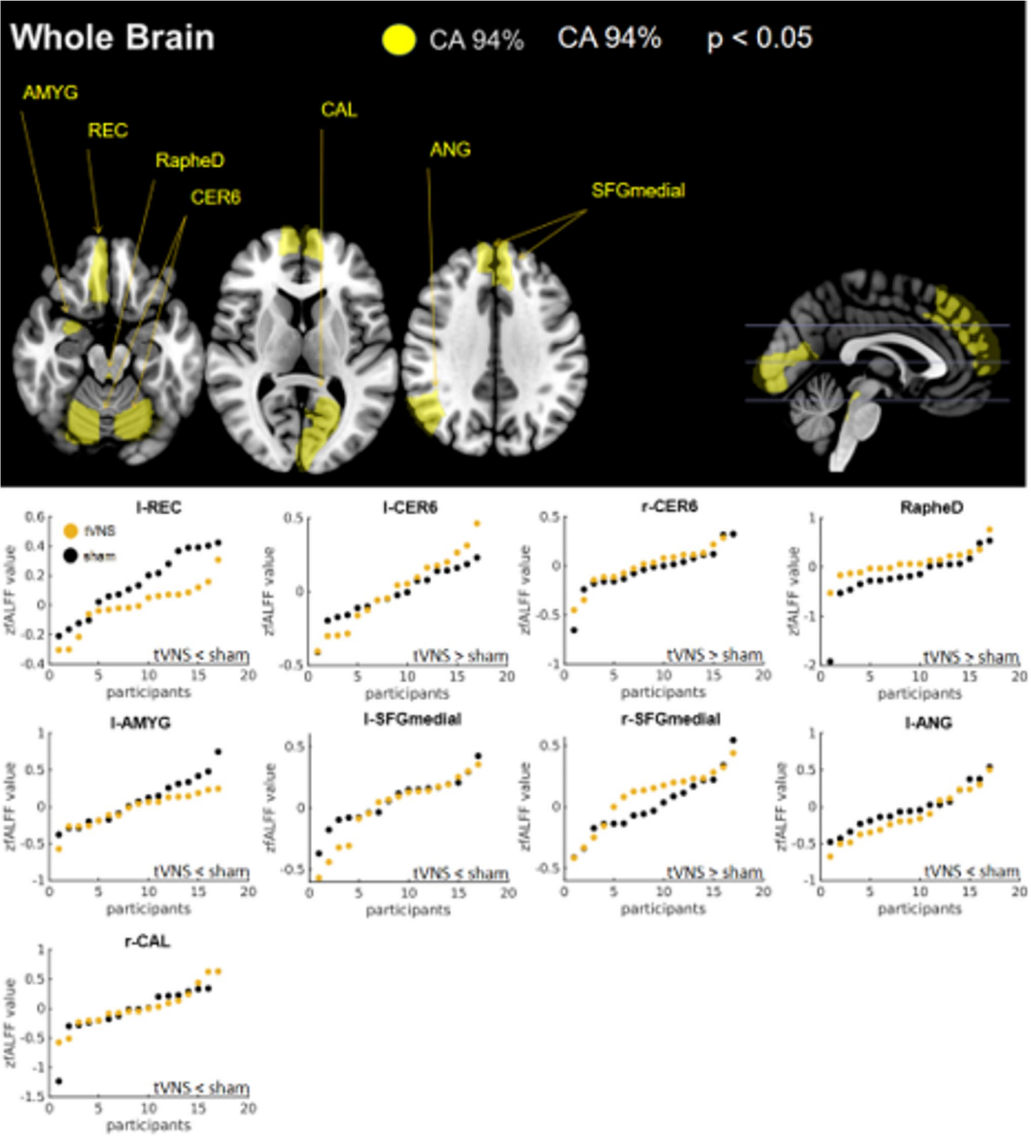
Classification result of the whole brain analysis. Upper panel: Shows the network of regions of interest (ROIs) selected by sequential forward floating selection algorithm (SFFS) out of 127 ROIs reaching a classification accuracy (CA) of 94 % by utilizing support vector machine (SVM) algorithm; p describes the probability that the classification result statistically equals the classification result of the control permutation (significance test). Lower panel: Visualization of mean zfALFF values and group differences per ROI for treatment groups (tVNS vs. sham). Impact of ROIs on CA is shown in a hierarchical ascending ordered from top-left to bottom-right: rectus gyrus (REC), cerebellum lobule 6 (CER6), dorsal raphe nuclei (RapheD), amygdala (AMYG), superior medial frontal gyrus (SFGmedial), angular gyrus (ANG), Calcarine sulcus (CAL); l = left, r = right

Participants were correctly allocated to their treatment condition with 94.1 % accuracy based on a subset (best subset), shown in Fig. 4, comprising 9/127 ROIs including l-Rectus Gyrus (REC), l- and r-Cerebellum Lobulue 6 (CER6), bilateral dorsal raphe nuclei (RapheD), l-Amygdala (AMYG), and r-superior medial frontal gyrus (SFGmedial), l-Angular Gyrus (ANG) and the r-Calcarine Sulcus (CAL; in a hierarchical order). The experimental CA was significantly different from both control CAs and the effect can be classified as very large (d_z_ >1.0, Table 4.b.). Moreover, this subset achieved a high sensitivity and specificity (1 and 0.9 respectively). Regarding these regions, tVNS increased the neural activity in the r-SFGmedial, the l-AMYG and in b-CER6 and b-RapheD regions but decreased the activity in the l-REC, l-ANG, l- SFGmedia and r-CAL (see Table 5B).

To evaluate the predictive value of these subset regions on hungriness ratings, a multiple (stepwise) regression analysis was performed. The analysis revealed a statistically significant model (*p* < 0.05) that comprised 5/9 ROIs of the subset and explained 45.4 % of the variation in hungriness ratings (see Table 6). Moreover, within this model, a significant interaction between r-SFGmedial * hungriness* treatment was found (see Fig. 6A). The interaction reflects that as treatment changes from sham (0) to tVNS (1) in combination with an increase in r-SFGmedial activity, the hungriness rating increased by 10.96 units. By inserting the group median values (Table 5B) of the r-SFGmedial activity into the model equation, it was shown that the subjects who received tVNS felt hungrier (1.7) post-treatment than those who received the sham stimulation (-0.3). Removing the interaction term from the model reduced the predictive value by 22.1 % emphasising the relevance of the treatment effect. However, no influence on the perceived satiety could be found for this subset.

**Table.6:** tVNS' effect on interactions between subset ROIs and hungriness / satiety rating

subset of whole brain analysis							
*response variable*	*Model*			*adj. R2*	*F(df*_*pred*_,*df*_*res*_)	*p*	
hungriness rating	~ l-REC + r-CER6. + l-SFGmedial + r-SFGmedial + l-ANG. + treatment + r-SFGmedial * treatment			0.31	* F(7,26) =* 3.09	0.017*	
*significant effects*	*β*	*p*					
r-CER6	5.47	0.022*					
l-ANG	-4.48	0.001**					
l-REC	-5.60	0.091°					
l-SFGmedial	-6.75	0.086°					
r-SFGmedial * treatment	**10.96**	0.022*					
satiety rating	~ l-REC + r-CER6 + l-SFGmedialc + r-SFGmedial + l-ANG + l-CAL + treatment + l-SFGmedial * treatment + r-SFGmedial * treatment + l-CAL * treatment			0.16	* F(10,23*) = 1.61	0.167 n.s.	
subset of the salience network analysis							
hungriness rating	~ r-aPFC + l-dACC + treatment			0.13	* F(3,30) =* 2.68	0.065°	
satiety rating	~ l-aINS + r-aINS + treatment + l-aINS * treatment + r-aINS * treatment			0.29	* F*(5,28) = 3.71	0.011*	
*significant effects*	*β*	*p*					
l. aINS	5.46	0.077°					
l. aINS *treatment	**16.53**	0.024*					
r. aINS * treatment	**-12.97**	0.043*					

postdiction^!^ withhierarchical stepwise multiple regression analysis

! postdiction refers to the circumstance that the response variables were measured before the measurements of the brain activity were conducted (approx. 3 h before). In both measurements, however, participants were in a fasted state.

Abbr: treatment = tVNS or sham stimulation, l- left, r- right, CER6 = cerebellum lobules 6, SFGmedial = superior medial frontal gyrus, CAL = Calcarine Sulcus, aPFC = anterior Prefrontal cortex, dACC = dorsal anterior cingulate cortex, aINS = anterior insula

Signif. codes: * p < 0.05, ° p < 0.1 (trend effect), n.s (not significant) p ≥ 0.1

### Network analysis

**Fig. 5:**
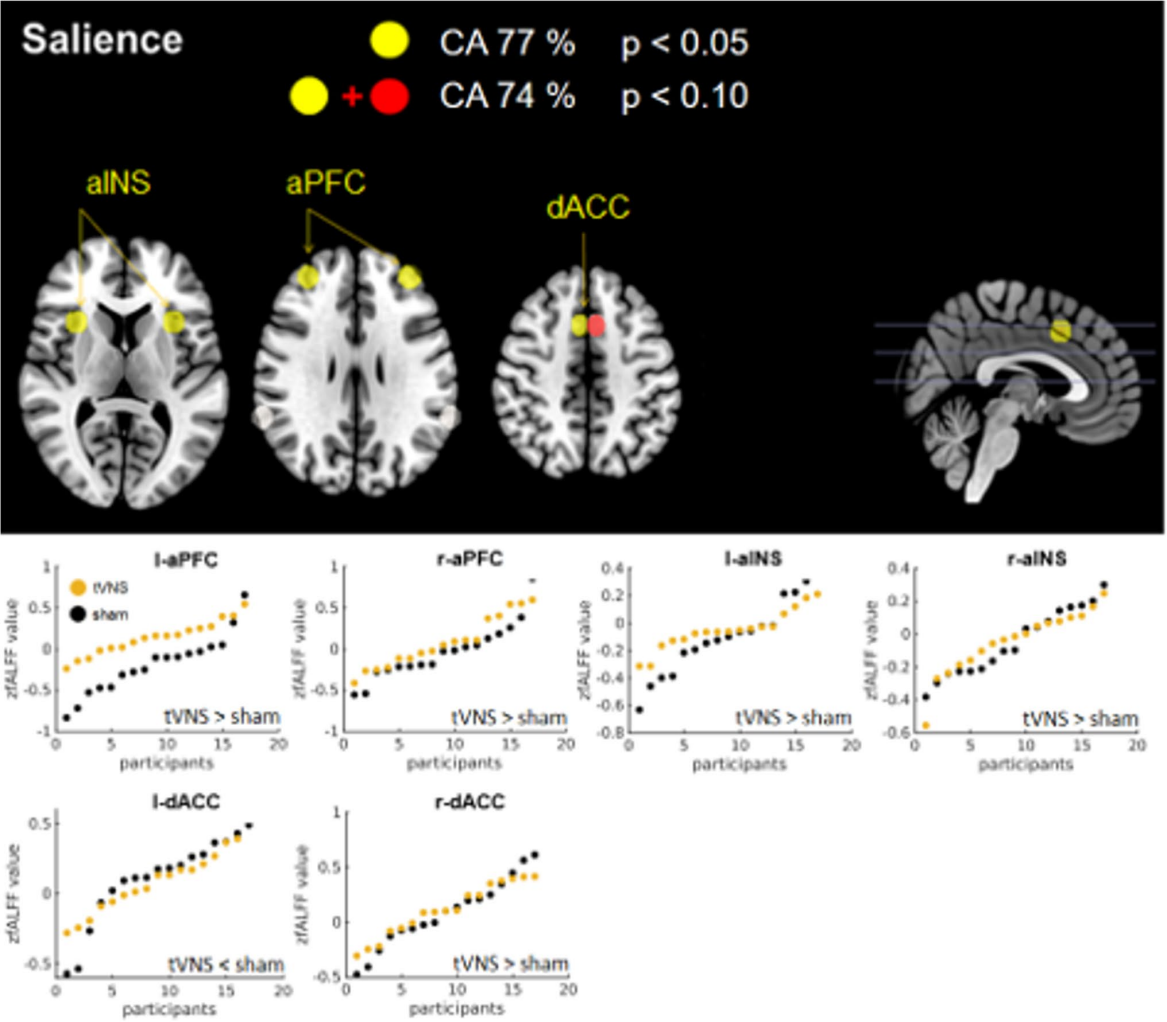
Classification result of the network analysis. Upper panel: Shows the network of regions of interest (ROIs) selected by sequential forward floating selection algorithm (SFFS) out of 8 ROIs by utilizing support vector machine (SVM) algorithm. Classification with yellow ROIs reached a classification accuracy (CA) of 77 % and with the additional red ROI a CA of 74 %; p describes the probability that the classification result statistically equals the classification result of the control permutation (significance test). Lower panel: Visualization of mean zfALFF values and group differences per ROI and stimulation group (hierarchical ascending ordered from top-left to bottom-right): anterior prefrontal cortex (PFC), anterior insula (aINS), dorsal anterior cingulate cortex (dACC)

A CA above 75 % was only reached for the SAL network (Table 3C1) and therefore tVNS did not affect the DA, REW, CE and VIS network in our study (see Table 4.c1-c4, respectively). A subset of the SAL network yielded a CA of 76.5 % (Fig. 5) and differed significantly from the result of both controls (Table 4.c1). It also showed a good classification quality as clarified by the sensitivity and specificity. This subset comprised 5/8 ROIs including the l-anterior prefrontal cortex (aPFC) and the r-aPFC, the l-anterior Insula (aINS) and the r-aINS as well as the l-dorsal anterior cingulate cortex (dACC; hierarchical order) but not the parietal regions. Regarding these ROIs, tVNS showed enhanced activity in both aPFC and Insula ROIs but decreased activity in the l-dACC (see Table 5C). The classification comprising the whole network achieved a CA of only 62 %.

**Fig. 6:**
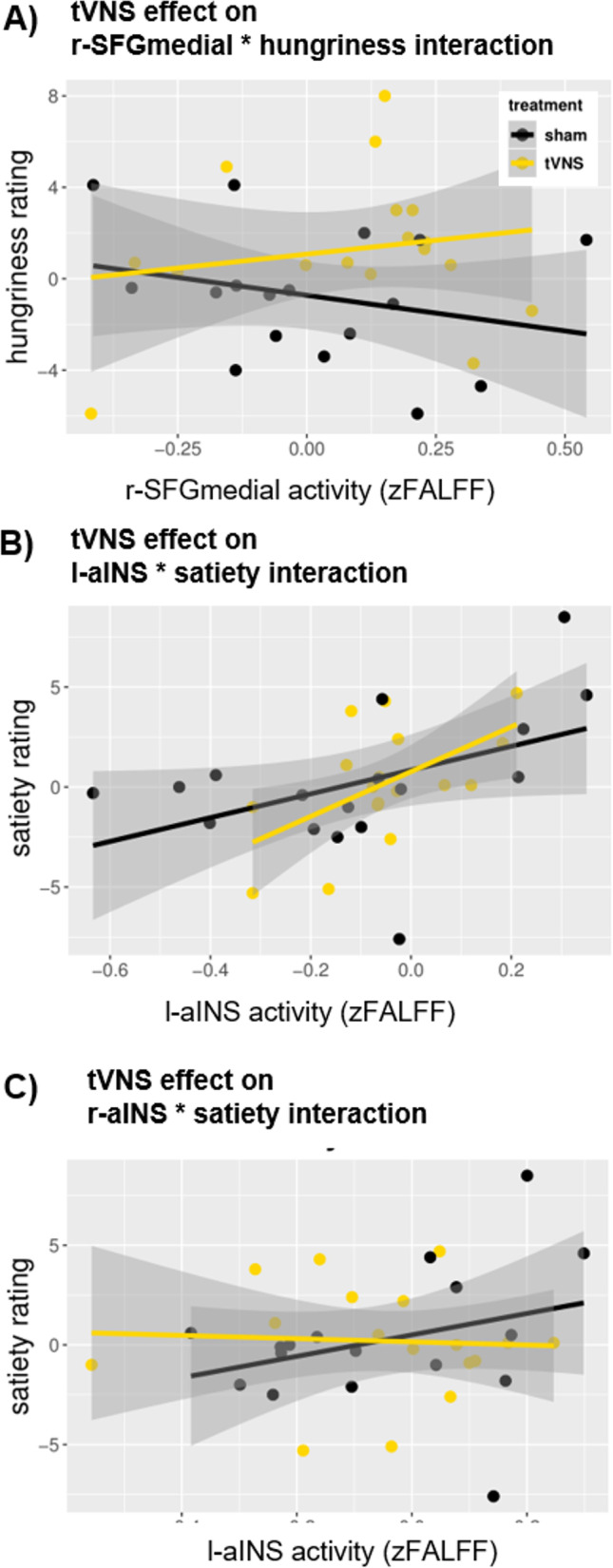
tVNS effect on the interaction between neural activity and the perception of feeding status. The found significant subsets revealed by Machine Leaning Classification were subjected to stepwise multiple regression including the stimulation treatment as interaction term. (**A**) shows the significant interaction based on the subset of the whole brain analysis. (**B) **and** (C**) show the significant interactions based on the salience network. Superior medial frontal gyrus (SFGmedial), anterior insula (aINS)

The regression analysis carried out to investigate the relationship between the regions of the significant subset and satiety ratings revealed a significant model (*p* < 0.05, see Table 6) that included 2/5 ROIs and elucidated 39.9 % of the variance in satiety. Within this model, significant interactions with the treatment (ROI* satiety * treatment) were found for both ROIs - the l- and r-aINS. (see Fig. 6B). The interactions show that as treatment changed from sham (0) to tVNS (1) the satiety rating increased by 16.9 units regarding the activity in the l-aINS but decreased by -13 units regarding the activity in the r-aINS. Based on this result, we calculated post-hoc the (absolute) hemispheric asymmetry (ROI right – ROI left, oriented on EEG conventions; (Smith et al., 2017; Stewart et al., 2014; Sutton & Davidson 1997) for each participant. We found that participants in the tVNS condition showed a higher asymmetry (median 0.03) as those who received the sham stimulation (median = -0.01). Here, positive values indicate a higher activity in the right hemisphere. This group-difference was, however, not significant (w = 136, *p* = 0.786). However, regression modelling revealed a significant relation between the insula-asymmetry with satiety ratings in the tVNS (R2 = 0.41, F[1,15] = 10.56, *p* = 0.005) but not in the sham group (R2 = 0.08, F[1,15] = 1.26, *p* = 0.279). Entering the median asymmetry values into the regression models showed that the asymmetry led to a higher satiety perception in the tVNS group (-0.99 units) compared to the sham group (-1.65 unit). However, investigating the predictive value of the subset ROIs on hungriness ratings yielded a model that reached statistical significance only at the trend level (see Table 6) and did not include any further treatment interactions.

## Discussion

The present study used an objective machine learning classification approach to assess whether five weeks of intermittent tVNS led to a modulation of rs-fMRI activity. Moreover, it was tested whether the regions distinguishing between tVNS and sham stimulation were related to the degree of hunger and satiety. Both points can be affirmed: Conditions could be classified reliably based on both, fALFF patterns in subcortical nuclei and a set of regions revealed in an analysis using 127 ROIs covering the whole brain. Moreover, in the tVNS condition a correlation between fALFF in a variety of regions and hunger and satiety ratings was obtained.

The classification on subcortical nuclei had a 79 % accuracy and was driven by increased activity in r-SN, r-NTS and PAG and a reduced activity in l-sTN. In other words, the subcortical regions that have been reported by Frangos et al. (2015) during acute stimulation were still changed in their rs activity 12 h after the last stimulation. This suggests that intermittent tVNS modulates spontaneous brain activity in a lasting fashion. This may be the neural basis of the beneficial effects of (t)VNS in conditions such as epilepsy and depression. It may also underlie potential effects of (t)VNS on body weight and metabolic parameters.

### Exploratory whole brain analysis

In the exploratory analysis we found a subset that correctly classified 94 % of the participants to their groups and that with a very high precision (sensitivity = 1, specificity = 0.9). Since this subset significantly differed from the performance of the both the traditional and our improved control permutation that controlled (additionally) the feature selection process, it can be generalized that tVNS had a modulating influence, especially on these brain regions. The classifiers performance was based on an increased activity measured in the ROIs of the bilateral CER6, bilateral RapheD, r-SFGmedial as well as on a decreased activity in l-REC, l-AMYG, l-SFGmedial and l-ANG and the l-CAL in the tVNS condition compared to control. It is noteworthy, that previous fMRI activation studies have also found tVNS effects on RapheD, CER6, ANG and AMYG (Yakunina et al., 2018) during acute stimulation. Again, this points to a prolonged effect of intermittent tVNS outlasting the stimulation by at least 12 h.

A further explorative analysis of the present data-set revealed a positive relationship between hunger ratings and r-SFGmedial activity. As pointed out above, r-SFGmedial was among the regions driving classification. A hypothesis that could be tested in future study would be that the modulation of the r-SFGmedial by tVNS modulates control of food intake. The medial frontal gyrus has been implicated in cognitive control in a variety of contexts ranging from bilingualism (Stasenko et al., 2020) to mindfulness training (Taren et al., 2017) and may thus be considered a part of the generic executive control network.

### Salience network

We also found preliminary evidence that tVNS influenced the salience network, as almost the entire network (5/8 ROIs) provided relevant information for a 76 % correct prediction of group membership. Compared to the control stimulation, tVNS showed in all subset ROIs an increased neural activity. An increased insula activity in regard to tVNS has been reported previously (Frangos et al., 2015) during acute stimulation. The salience network has been shown to play a role in emotional control and the processing of interoceptive states. Moreover, it switches between the default mode network and the central executive network (Goulden et al., 2014; Sridharan et al., 2008; Terasawa et al., 2013). It has been implicated in depression (Ellard et al., 2018; Han et al., 2020; Nolen-Hoeksema et al., 2008). Perceptual salience also plays a major role in the decision making of food choices as experimentally demonstrated by Dai, Cohn and Moher recently (Dai et al., 2020). Modulation of the salience network by tVNS might therefore underlie the effects in depression and – possibly – food intake.

In line with this notion, the regression analysis revealed significant treatment interactions of both aINS regions with satiety ratings. The aINS (together with the dACC and medial PFC) is suggested to be one of the core areas with regard to the processing of interoception - the sense of the internal state of the body (Uddin et al., 2017).

Obese people compared to lean people showed reduced insula activity in resting state neural activity in a pre-meal fasted state (Kennedy & Dimitropoulos, 2014). The greater bilateral insula activity shown in our study could indicate that tVNS could reverse obesity-associated changes in the processing of interoceptive signals.

This suggestion is supported by the result of our post-hoc analysis that showed a stronger right-sided functional asymmetry in the tVNS group regarding the insula cortices. The processing of interoception has been suggested to happen in a right-lateralized body-awareness network since in particular the right anterior insula cortex is highly interconnected with primary visceral and somatosensory areas such as posterior insula and somatosensory cortex (Cerliani et al., 2012; Chang et al., 2013). In our study, the higher right-sided asymmetry in the tVNS group significantly predicted the corresponding satiety rating and this relationship was not found for the lower asymmetry in the sham group.

### Limitations

The results of machine learning analyses depends on the used components and the specific algorithms; Using a different classifier type (i.e., Support Vector Machine Regression, Bayesian Net) in combination with a different validation strategy could possibly lead to a different result. Therefore, future studies should investigate neural tVNS effects with other machine learning approaches, to validate our findings.

Another aspect concerning generalizability of the results is the problem of overfitting. Data overfitting means that the machine learns the classification pattern based on noise instead of real information affecting the generalizability of the classification result. In our analysis, we improved the traditional permutation test procedure by including the feature selection process resulting in much more conservative results (compare results of traditional PC and FSC in Table 4). In the FSC only three subsets yielded significance. These subsets showed a control CA above 50 % reflects the extent of overfitting.

reflects the extent of overfitting. However, subtracting the estimated amount of overfitting from the experimental CA, approx. 7 %, 9 % and 12 % remain for the Brainstem, whole brain, and salience subset, which probably reflect the true and noise-purified effect of the tVNS treatment.

## Data Availability

Data will be provided at www.datadryad.org for fee download.
